# The effects of family education program on the caregiver burden of families of elderly with dementia disorders

**Published:** 2010

**Authors:** Saeed Pahlavanzadeh, Fatemeh Ghaedi Heidari, Jahangir Maghsudi, Zahra Ghazavi, Saeed Samandari

**Affiliations:** * Department of Psychiatric Nursing, School of Nursing and Midwifery, Isfahan University of Medical Sciences, Isfahan, Iran; ** School of Nursing and Midwifery, Isfahan University of Medical Sciences, Isfahan, Iran; *** Associate Professor, Psychiatric for Elderly, Modarres Psychiatric Hospital, Najafabad, Isfahan, Iran

**Keywords:** Education program, caregiver, dementia, disease burden

## Abstract

**BACKGROUND:**

Family caregivers are an essential part of health care services for elderly with dementia disorders, because of providing care for such patients is a big burden for their families. This study aimed to assess the effects of family education program in reducing the burden of families of elderly with dementia.

**METHODS:**

This was a clinical trial, in which 50 family caregivers of the elderly patients with dementia who had referred to two referral centers for dementia in the city of Isfahan were selected with convenient sampling method and were randomized to experimental and control groups. The experimental group participated in a family education program but the control group did not. Data were collected by Zarit’s caregiver burden scale completed by caregivers of both groups before, right after, and one month after family education program. Also, Mini-Mental Status Examination was conducted for elderly before the program. Finally, data were analyzed in SPSS software.

**RESULTS:**

Caregivers’ burden was gradually increased in the controls, but decreased in the experimental group during the study. The means of caregivers’ burden before, right after, and one month after family education program were 42, 35.44, and 33.56 in the experimental group, respectively, while they were 43.28, 46.8 and 50.64 in the control group, respectively. Also, there was a significant difference between caregivers’ burden of the two groups immediately after intervention, but one month after intervention no significant difference was seen between the two groups.

**CONCLUSIONS:**

Since conducting this program could reduce caregivers’ burden of families of elderly with dementia, developing such programs and evaluating them within research projects are recommended.

Dementia is a common psychonervous disorder among elderly that has destructive effects on patients’ cognition, perception, language, behavior and motional abilities.^1^ This disease gradually destroys the ability of problem solving and learning new skills.^2^ According to the World Health Organization statistics, there are 24.3 million people around the world currently suffering from this disease and 4.6 million new patients are added to this number annually.^3^ In Iran, dementia disorders are not screened yet, but the World Alzheimer Society estimates that about 250,000 to 300,000 people are suffering from this disease in Iran.^4^ As the population of elderly is increasing in the world, it is expected that the number of those suffering from this disorder increase as well.^5^,^6^ Usually, dementia disorders have progressive process,^1^,^7^ so that patients would need care at some point; because they lose the ability to take care of themselves to the extent that they need care and support 24 hours a day.^8^,^9^ This care is offered mainly at home^8^,^10^ and by family members or patients’ friends or caregivers.^11^

Taking care of these patients is one of the most problematic and challenging caregiving conditions;^12^ so that such caregivers are called “second victims of dementia”.^13^ In general, tak-ing care of these elderly patients is associated with tension and, as a result, various complications^14^,^15^,^16^ that are described as caregiving burden, which includes physical, emotional, financial and social problems.^17^

The effects of dementia disorders are not limited to the patients and expands to their family and friends.^18^ Therefore, it is necessary to allocate part of care programs to families.^7^ Caregivers need education about disease and care methods.^19^ In some countries, various supportive and educational programs are offered for families of patients with dementia and many studies have evaluated these programs. In Iran also, there are a few studies to determine the caregivers’ burden to prepare proper educational programs for them. The study of Zahri et al aimed to find problems of caregivers of patients with Alzheimer, who referred to Tehran Alzheimer Association and most caregivers described their problems as very much and severe. The most common problems included making relationship between patient and other members of the family, sleep disorders, and exhaustion.^20^ Considering the lack of researches on the topic in Iran and the difficulty of caregiving of dementia patients, the present study aimed to determine the effects of family educational program on caregivers’ burden.

## Methods

This clinical trial conducted on 2009. The study population included caregivers of elderly with dementia living in the city of Isfahan. Sampling was based on convenience method. Inclusion criteria were as being the main caregiver and having the responsibility to take care of the patient, age of more than 20 years, education level of above elementary school, caregiving just to the patient in the family, not attending any other family educational sessions before that, and not sharing caregiving with others. Also, exclusion criteria included attending less than 4 sessions of the educational program, patient’s death during the study, suffering from severe and chronic physical or mental disorders that can be obstacle to caregiving, drug abuse, and providing longterm institutional care for patient during the study.

In this study, sample size was calculated 50 caregivers considering similar studies^21^ and by statistical formula. Considering the probability of losing of samples during the study, 60 samples were calculated. Sampling was conducted from April to July 2009, by referring to Modares Psychiatric Hospital and Shariati Psychiatric clinic in the city of Isfahan, which are the centers for elderly with dementia, and the samples were selected from caregivers of the elderly referring to these centers. The samples were randomly divided into two groups of case (n = 30) and control (n = 30). Caregivers of the case group attended family educational program after signing a consent form. Patients and controls were not present in this program. The content of the program was based on an educational pamphlet prepared and edited by the researchers through reading books and articles. The content was approved by 10 psychiatrists, neurologist and faculty members of the School of Nursing and Midwifery of Isfahan University of Medical Sciences.

This program included 5 weekly sessions^22^ using lecturing, group discussions (10-15 members in groups), and question and answer as table 1 presents. The duration of each session was 90 minutes^23^ and the place was Noor Hospital in Isfahan. This program was conducted under the supervision of the first researcher who has several years of experience as a university lecturer and patients and their families’ educator and also has a master degree in psychiatry. Meanwhile, CDs of all the sessions were provided to caregivers of the case group. In this study, the diagnosis of dementia was based on Diagnostic and Statistical Manual of mental disorders-IV-Text Revised (DSM-IV-TR) and by a psychiatrist specialist in the elderly through structured clinical interview. Also, to assess the cognitive condition of elderly, Mini Mental status Examination^24^ was used. The validity and reliability of this test are approved by other studies using concurrent criterion and Cronbach’s alpha by splitting (α = 0.71-0.78, r = 25).^25^–^27^ This test includes 30 items and assesses some cognitive functions such as direction finding, recording information, attention and calculation, remembering and language skills. To collect data of caregivers, Zarit Caregiver Burden Scale (ZCBS) was used.^28^ The validity of this scale was assessed by using books, resources and modeling this scale as well as opinions of the professional faculty members of the university. The reliability of this scale was approved by previous studies through re-test with amount of 94% (r = 29).^29^,^30^ This scale includes 22 items and measures the mental burden of caregivers. This scale was completed before, right after, and one month after the educational program by both groups. Collected data were analyzed using SPSS software version 16 and descriptive statistics (frequency and mean) and inferential statistics (t-test, ANOVA, and chi-square test).

**Table 1:** Family education program sessions

**Session 1**	Changes in the elderly, the definition, stages and symptoms, risk factors, diagnostic methods, and treatment of dementia
**Session 2**	Ways to improve communication with patients, feeding them, and ways to control and deal with their urine and fecal incontinence, methods to improve their sleep, bathing and personal hygiene, and dressing the patient
**Session 3**	Methods to control patients’ unusual behavior including repetitive behavior, pursuing the caregiver, shouting, unreasonable laughing and crying, and disregarding social rules
**Session 4**	Methods to control patients’ unusual behaviors including excessive walking and restlessness, hiding things, being suspicious and slandering, irrelevant resistance, and stubbornness
**Session 5**	Methods to control patients’ unusual behavior including vagrancy and wandering and aggression, safety measures at home, how to entertain patients at home, and methods of reducing caregivers’ burden

The ethical committee of Isfahan University of Medical Sciences approved the study.

## Results

The mean age in the study and the control out of 60 pairs of caregiver-patient who entered the study, 5 subjects in the case group and 5 subjects in the controls were excluded due to lack of presence in educational sessions and also, not being available right after the program and one month after it. Data analysis was done on the remaining 50 pairs of caregiver-patient. The results of the chi-square and independent t-test showed that the two groups of case and control were almost matched in variables such as age, gender, marital status, educational level of caregivers and patients, the duration and severity of patients’ disease, duration of caregiving, and family relationship between patients and caregivers. These variables are presented in table 2.

**Table 2:** Demographic data of patients and caregivers in the two groups of case and control

	Patients		Caregivers	

	Case n = 25	Control n = 25	Case n = 25	Control n = 25
**Age (years)**	72 (8.91)	67.72 (11.84)	42.88 (15.13)	46.56 (14.51)
**Gender**				
Men	56%	44%	16%	32%
Women	44%	56%	84%	68%
**Marital status**				
Single	0%	0%	16 %	0%
Married	68%	80%	84%	0%
Widowed	24%	0 %	0%	0%
Divorced	8%	20%	0%	0%
**Education level**				
Illiterate	48%	16%	0%	0%
Primary school	32%	68%	48 %	72%
Guidance school	4%	8%	8%	8%
High school diploma	16%	8%	24%	20%
Higher education	0%	0%	20%	0%
**Duration of illness (years)**	21.2 (5.1)	2.65 (1.4)		
**Severity of illness (MMSE)**	14 (3.38)	13.44 (2.78)		
**Caregiving duration (years)**			2.17 (1.41)	2.39 (1.32)
**Relationship between patient and caregiver**				
Spouse			28%	48%
Child			40%	36%
Sister			4%	0%
Other			28%	16%

Study results showed that caregivers’ burden in the case group gradually decreased and the mean (SD) of caregivers’ burden scores before, right after, and one month after the educational program were 42 (65.17), 44.35 (65.15) and 56.33 (54.14), respectively. Comparing these means using repeated measures ANOVA showed a significant difference between the means of this group (p < 0.001). Moreover, paired t-test showed a significant difference between means of caregivers’ burden scores before and right after the intervention and also before and one month after the intervention (p < 0.001). However, there was no significant difference between the mean scores right after and one month after the intervention.

The findings showed that caregivers’ burden in the control group was gradually increased and the mean (SD) scores of before, right after, and one month after the intervention were 43.28 (13.08), 46.8 (3.13) and 64.50 (25.13), respectively. Comparing the means in this group using repeated measures ANOVA showed a significant difference (p < 0.001). Moreover, paired t-test showed a significant difference between means of caregivers’ burden scores before, right after, and one month after the intervention and also right after and one month after the intervention (p < 0.001).

Comparing the caregivers’ burden scores of the two groups of case and control before, right After, and one month after the intervention using independent t-test showed that before the intervention there was no significant difference between the two groups and both groups were the same at that time. However, the difference between the two groups right after was significant (p < 0.001); but one month after intervention no significant difference was seen between the two groups.

## Discussion

As the results of the present study showed, most caregivers were women. In Iran, caregiving to patients, especially elderly, is usually by women. Studies of other countries also showed that women in most cases are the main caregiver of patients in the family. Usually the caregivers are middleage, married women.^31^ In a study by National Alliance for Caregiving (NAC) and American Association of Retired Persons (AARP), 61% of caregivers were women with a mean age of 46 years.^32^ These findings are similar to those of the present study.

The present study showed that caregivers’ burden in the case group was significantly reduced right after the family educational program. This result was not found in any other studies reviewed.^22^,^23^,^33^,^34^ In all of these studies, caregivers’ burden was reduced right after the educational-supportive intervention, but this reduction was not significant compared with before the intervention. This difference in results can be to some extents related to study method, especially the components of the program including educational contents. Another factor is the educational-supportive facilities accessible to caregivers at the research environment. Unfortunately, such facilities were not available for caregivers in Isfahan, but other studies were in the countries where such facilities were available to facilitate caregiving and keep the caregivers’ burden low, which can downsize the effects of the research intervention.

Also, the results of the present study in the case group showed the continuity of the positive effects of the program on the caregivers’ burden during one month after the intervention; but due to the effects of the increasing severity of cognitive disorder during the time of study plus the increase of patients’ problems during this time, the difference between caregivers’ burden right after and one month after the intervention was not significant. This finding agrees with the results of similar studies.^22^,^35^,^36^

Findings showed that the caregivers’ burdens in the control group were gradually increased during the study and there was a significant difference between the caregivers’ burden in this group before, right after, and one month after the intervention. Lack of supportive educational facilities along with increasing severity of cognitive disorder can be the cause of this increase in caregivers’ burden. However, the study of Dias et al in India, which aimed to determine the effectiveness of a homecaring program to support caregivers of dementia patients, revealed that caregivers’ burden in the control group 3 months after the intervention showed a little reduction compared to that right after intervention.^35^ In other studies, caregivers’ burden was increased by time, but the difference between caregivers’ burden at different times of the study was not significant.^22^,^23^,^33^,^34^,^36^

Findings also showed a significant difference between caregivers’ burden of the two groups in all times of the study except before the intervention. This finding is similar to two other studies,^22^,^36^ but dissimilar with several other studies due to different methods and educational facilities provided for caregivers in the study environment.^23^,^33^–^35^

A significant feature of the present study was providing caregivers constant access to the educational sessions by preparing CDs of those sessions. Also, considering the results of the study, it supports inclusion of mental educational programs among necessary cares for caregivers of the dementia patients. In addition, researchers believe that one of the causes for the relative success of this program is the contents, which were prepared considering the determined problems of the similar studies. Moreover, the present study used group teaching methods, which are more effective than private teachings. The main limitation of the study was short-term followup that were decided because of limited term of education and probability of losing too many samples. Therefore, it is suggested to perform this program with longer term follow-up and evaluation.

It can be concluded that the results of the present study generally proved the positive effects of the family education program on caregivers’ burden of the dementia patients and as a result decreased this burden.

The authors declare no conflict of interest in this study.

## References

[1] Keltner NL, Schwecke LH, Bostrom CE (2003). Psychiatric nursing.

[2] Varcarolis EM (2004). Manual of psychiatric nursing care plans: diagnosis, clinical tools and psychopharmacology.

[3] (2006). World Health Organization. Neurological disorders: public health challenges.

[4] Alzheimer’s Association of Iran. About dementia.

[5] Evans K, Elder R, Nizette D (2004). Psychiatric mental health nursing.

[6] Craven RF, Hirnle CJ (2003). Fundamentals of nursing: human health and function.

[7] Eby L, Brown NJ (2005). Mental health nursing care.

[8] Ebersole P, Hess P, Toughy TA, Jett K, Luggen AS (2005). Gerontological Nursing and Healthy Aging.

[9] Magliano S Families of people with severe mental disorders: difficulties and resources.

[10] Ebersole P, Touhy TA (2006). Geriatric nursing: growth of a specialty.

[11] Wiles J (2003). Informal caregivers’ experiences of formal support in a changing context. Health Soc Care Community.

[12] Papastavrou E, Kalokerinou A, Papacostas SS, Tsangari H, Sourtzi P (2007). Caring for a relative with dementia: family care-giver burden. J Adv Nurse.

[13] Neal LJ, Guillett SE (2004). Care of the adult with a chronic illness or disability: a team approach.

[14] Bradley PJ (2003). Family caregiver assessment. Essential for effective home health care. J Gerontol Nurs.

[15] Kim JS, Lee EH (2003). Cultural and noncultural predictors of health outcomes in Korean daughter and daughter-in-law care-givers. Public Health Nurse.

[16] Sawatzky JE, Fowler-Kerry S (2003). Impact of caregiving: listening to the voice of informal caregivers. J Psychiatr Ment Health Nurse.

[17] Nasso J, Celia L (2007). Dementia care: in-service training modules for long term care.

[18] Dinkins RL (2004). The effect of support group on the self-efficacy of caregivers of Alzheimer’s patients.

[19] Videbeck SL (2010). Psychiatric mental health nursing.

[20] Zohari S, Khatooni S, Abed Saeedi Zh, Alavi Majd H, Yaghmai F (2006). Review the main problems of carers of patients with Alzheimer’s disease Alzheimer’s Association referred to Tehran. Journal of Nursing and Midwifery.

[21] Lindstrom Bremer MK (2007). Partners in care-giving: effects of psycho educational intervention on Alzheimer's care-giving daughters. [PhD Thesis].

[22] Andren S, Elmstahl S (2008). The relationship between caregiver burden, caregivers’ perceived health and their sense of coherence in caring for elders with dementia. J Clin Nurs.

[23] Martin Carrasco M, Martin FM, Valero CP et al (2009). Effectiveness of a psycho-educational intervention program in the reduction of caregiver burden in Alzheimer’s disease patients’ caregivers. International Journal of Geriatric Psychiatry.

[24] Folstein M, Folstein S, McHugh P (1975). “Mini-mental state”. A practical method for grading the cognitive state of patients for the clinician. J Psychiatr Res.

[25] Frooghan M, jaafari Z, Shirin bayan P, ghaem Farahani Z, Rahghozar M (2008). Brief cognitive status examination standardization elderly in Tehran 2006. Advances in Cognitive Science.

[26] Nejati V, Ashayeri H (2006). The relationship between cognitive disfunction and depression in elderly. Iranian Journal of Aging.

[27] Sadock BJ, Sadock VA (2003). Synopsis of psychiatry.

[28] Zarit SH, Reever KE, Bach-Peterson J (1980). Relatives of the impaired elderly: correlates of feelings of burden. Gerontologist.

[29] Gort AM, Mingot M, Gomez X, Soler T, Torres G, Sacristán O (2007). Use of the Zarit scale for assessing caregiver burden and collapse in caregiving at home in dementias. Int J Geriatr Psychiatry.

[30] Taub A, Andreoli SB, Bertolucci PH (2004). Dementia caregiver burden: reliability of the Brazilian version of the Zarit care-giver burden interview. Cad Saude Publica.

[31] Harkreader HC (2000). Fundamentals of nursing: caring and clinical judgment.

[32] Ebersole P, Touhy T, Hess P (2008). Toward healthy aging: human needs & nursing response.

[33] Gitlin LN, Winter L, Burke J, Chernett N, Dennis MP, Hauck WW (2008). Tailored activities to manage neuropsychiatric behaviors in persons with dementia and reduce caregiver burden: a randomized pilot study. Am J Geriatr Psychiatry.

[34] Hébert R, Lévesque L, Vézina J, Lavoie JP, Ducharme F, Gendron C (2003). Efficacy of a psychoeducative group program for caregivers of demented persons living at home: a randomized controlled trial. J Gerontol B Psychol Sci Soc Sci.

[35] Dias A, Dewey ME, Souza JD, Dhume R, Motghare DD, Shaji KS (2008). The Effectiveness of a Home Care Program for Supporting Caregivers of Persons with Dementia in Developing Countries: A Randomized Controlled Trial from Goa, India. PLoS ONE.

[36] Gavrilova SI, Ferri CP, Mikhaylova N, Sokolova O, Banerjee S, Prince M (2009). Helping carers to care-the 10/66 dementia research group’s randomized control trial of a caregiver intervention in Russia. Int J Geriatr Psychiatry.

